# Supplementary material for: Association of Preoperative Copeptin Levels with Risk of All-Cause Mortality in a Prospective Cohort of Adult Cardiac Surgery Patients
Source: Cells. 2024 Jul 15;13(14):1197. doi: 10.3390/cells13141197 (PMC11274732; doi:10.3390/cells13141197)
Supplement: Supplementary file 1 [file cells-13-01197-s001.zip › cells-3063511-supplementary.pdf]

Supplementary Material

Supplementary Figure S1: Scatterplot of preoperative Euroscore II values (abscissa) with preoperative copeptin levels (ordinate). The fit of a linear regression of log10-transformed
Euroscore II levels (covariate) and log10-transformed copeptin levels is shown.

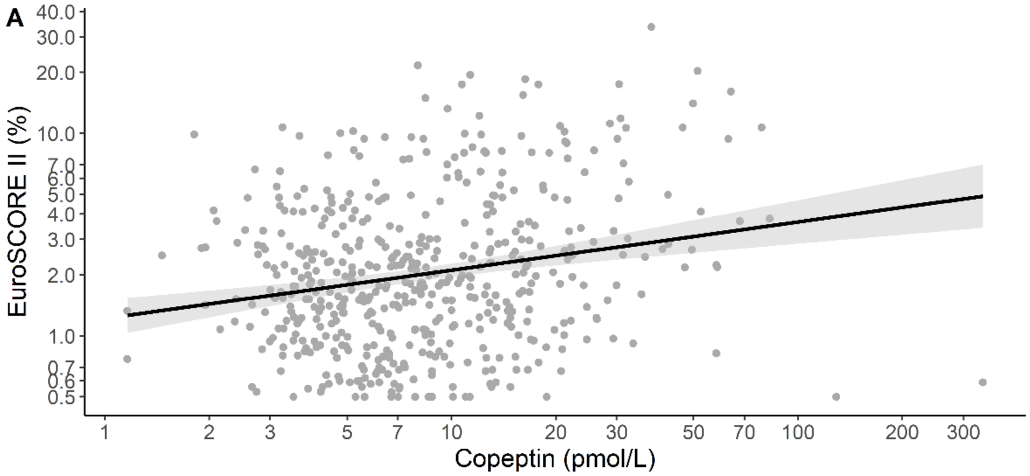

$Euroscore_{log10}$ = 0.09 (95%-CI: -0.1 to 0.18) + 0.24 (95%-CI: 0.14 to 0.33, $p<0.001$) x $Copeptin_{log10}$

Supplementary Figure S2: Kaplan-Meier curve for 1-year follow-up. The overall survival probability (black line) is displayed, as well as stratified survival probability according low (<15.9 pmol/L, blue line) and high (≥15.9 pmol/L, red line) preoperative copeptin levels.

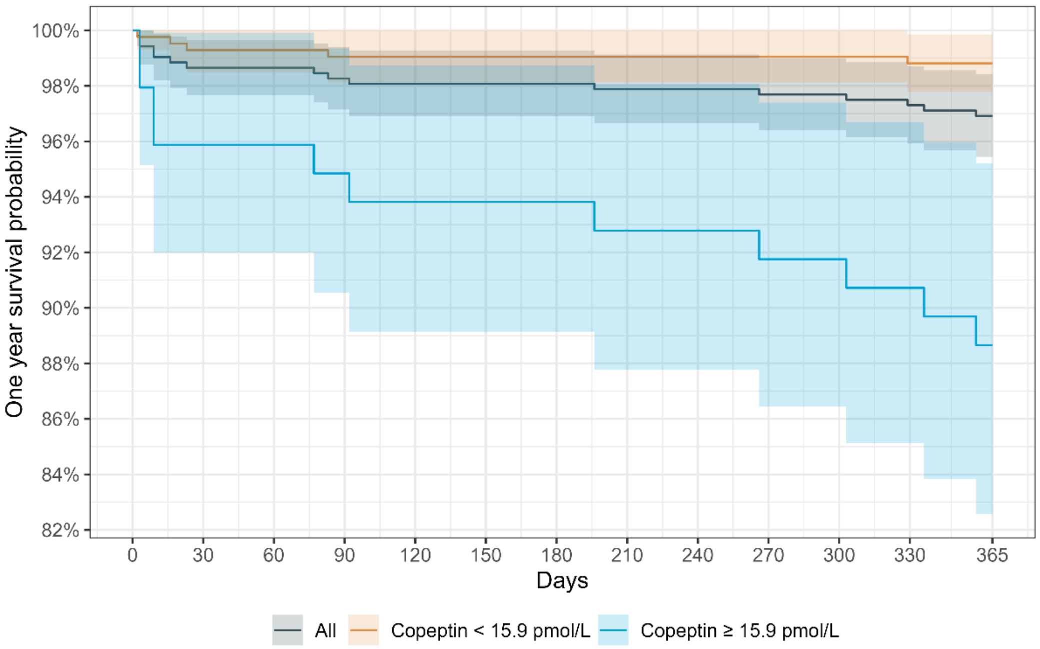

At Risk

	0	30	60	90	120	150	180	210	240	270	300	330	365
All	519	512	512	510	509	509	509	508	508	507	507	505	503
Copeptin < 15.9 pmol/L	420	417	417	416	416	416	416	416	416	416	416	415	415
Copeptin ≥ 15.9 pmol/L	97	93	93	92	91	91	91	90	90	89	89	88	86

Events

	0	30	60	90	120	150	180	210	240	270	300	330	365
All	0	7	7	9	10	10	10	11	11	12	12	14	16
Copeptin < 15.9 pmol/L	0	3	3	4	4	4	4	4	4	4	4	5	5
Copeptin ≥ 15.9 pmol/L	0	4	4	5	6	6	6	7	7	8	8	9	11
